# Novel Phonography-Based Measurement for Fetal Breathing Movement in the Third Trimester

**DOI:** 10.3390/s21010211

**Published:** 2020-12-31

**Authors:** Márton Áron Goda, Tamás Telek, Ferenc Kovács

**Affiliations:** 1 Faculty of Information Technology and Bionics, Pázmány Péter Catholic University, Práter utca 50/a, 1083 Budapest, Hungary; kovacs.ferenc@itk.ppke.hu; 2 St. Margaret Hospital, Bécsi út 132, 1032 Budapest, Hungary; drtelektamas@gmail.com

**Keywords:** fetal breathing movement (FBM), biophysical profile (BPP), phonography, ultrasound sonography, photogrammetry, FBM epoch, FBM episode

## Abstract

The detailed assessment of fetal breathing movement (FBM) monitoring can be a pre-indicator of many critical cases in the third trimester of pregnancy. Standard 3D ultrasound monitoring is time-consuming for FBM detection. Therefore, this type of measurement is not common. The main goal of this research is to provide a comprehensive image about FBMs, which can also have potential for application in telemedicine. Fifty pregnancies were examined by phonography, and nearly 9000 FBMs were identified. In the case of male and female fetuses, 4740 and 3100 FBM episodes were detected, respectively. The measurements proved that FBMs are well detectable in the 20–30 Hz frequency band. For these episodes, an average duration of 1.008 ± 0.13 s (*p* < 0.03) was measured in the third trimester. The recorded material lasted for 16 h altogether. Based on these measurements, an accurate assessment of FBMs could be performed. The epochs can be divided into smaller-episode groups separated by shorter breaks. During the pregnancy, the rate of these breaks continuously decreases, and episode groups become more contiguous. However, there are significant differences between male and female fetuses. The proportion of the episodes which were classified into minimally 10-member episode groups was 19.7% for males and only 12.1% for females, even at the end of the third trimester. In terms of FBM detection, phonography offers a novel opportunity for long-term monitoring. Combined with cardiac diagnostic methods, it can be used for fetal activity assessment in the third trimester and make measurement appreciably easier than before.

## 1. Introduction

Fetal breathing movement (FBM) is a peculiar phenomenon of intrauterine life. It is special because there is no oxygen uptake (which is normally called breathing); only the diaphragm contraction and relaxation are detectable [1]. According to the current conception of medicine, this is essentially a preparation for the time after birth, when the infants must breathe with great confidence.

During the contraction and relaxation of the diaphragm, the surrounding tissues and fluids are also moved, which creates sounds that can be recorded on the maternal abdominal wall with a suitable microphone. This means that the FBM can also be determined by a novel method using phonography, in addition to the usual ultrasonic measurement. The advantage of this is that the measuring device is definitely much simpler here, and this measurement is not time-dependent (i.e., the sonographer does not have to wait for the FBMs, which are relatively rare) because the passive phonogram recorder can be left on the maternal abdomen for a long time.

The method has an additional advantage that has not yet been fully developed. Phonocardiography has been used in several countries for nearly 20 years to measure fetal heart rate (FHR). Since the sound of the moving diaphragm occurs in a much lower frequency band than the heart sounds, in principle, it may be possible to measure the different types of fetal activity simultaneously and continuously, even in a home environment [2]. If these voices can be reliably separated, their relationship might also be explored, which is a very important field of current research. This would certainly be important for the fetus because the FBM requires a physical effort, and in this way, the measurements can provide more information about fetal well-being.

### 1.1. Former Research

For more than 40 years, ultrasound has been one of the most advanced examination methods in the field of fetal monitoring. Hitherto, these measurements could only be carried out under medical supervision for a limited length of time and primarily in a hospital. However, due to the large number of unexplained intrauterine fetal deaths occurring in the third trimester [3], the need to have assessment methods that are capable of fetal activity monitoring, even at home, has become apparent.

The existence of FBM has been known for more than 130 years from Preyer [4] and Ahlfeld [5], and its importance has also been confirmed by other studies of Adams et al. [6]. Initially, FBM measurements were conducted with simple mechanical sensors on the maternal abdominal wall. However, information obtained in this way is very poor. The first relevant information source about FBM was Patrick et al. [7], who identified the occurrence and duration of this kind of motion, distinguishing it from other fetal movements such as trunk rotation or head and limb movements. The study of Junge et al. [8] investigated the relationship between sleeping states and FBMs. The study of Noguchi et al. [9] explored the relationship between the two mentioned actions, but their results were not fully confirmed. With the development of ultrasound measurements, it has become possible to complete much more accurate examinations than before, an opportunity that was exploited by Andrews et al. [10] in their research.

Slowly, FBM has become an essential index of fetal health as a result of all these early measurements. This is indicated by the fact that the number and duration of FBMs have become the part of the biophysical profile (BPP), even though the precise cause of FBM has not been pinpointed. Later, the regular measurement of FBM also became a required test. However, initially, this examination was very difficult to carry out because of the random occurrence of the breathing movement and considerably extended the investigation time. Independently from each other, Berger et al. [11] and Talbert et al. [12] made efforts to solve this problem. They measured the signal of the mechanical movement with a piezo-transducer placed on the maternal abdomen. Another improvement was made by Goovaerts et al. [13] and Ansourian et al. [14], who measured the generated current on a coil formed on a flexible membrane while it was moving in the magnetic field on the mother’s belly. However, they were unable to obtain accurate information, such as the exact time function of the FBM.

Nevertheless, extensive research started to investigate fetal movement. The study of the relationship between FBM and fetal heart function came into view again with the work of Moczko [15] and Foulquiere [16]. Florido et al. [17] also made efforts to meet the challenges of this research topic, already using ultrasound measurement for this purpose. The field of FBM research appeared to be boosted thanks to the arrival of advanced signal processing techniques and included a variety of frequency and spectrum processing algorithms. The primary purpose of these was to detect the diseases occurring already at fetal age. In this respect, the work of Ansourian [14] and Dornan [18] played an important role in FBM research. Finally, the research of Cousin [19] and Akay [20] should be mentioned, which opened new perspectives in biomedical signal processing using the matching pursuit (MP) method. In relation to fetal movements, the issue of hiccups arose [21], as well as its relation to FBM and their differences [22]. Finally, a completely new question is now the possible link between FBM and sudden infant death syndrome (SIDS) [23]. The latest research by Shuffrey et al. [24] confirms significant activity differences between male and female fetuses, a finding which is also closely related to the present study.

### 1.2. Phonography-Based Research

In the past 20 years, there has been a significant change in FBM research with the appearance of phonocardiography, which has become an important tool for fetal heart function detection. However, there is a serious problem, namely that the identification of the very low-level heart sound is often disturbed by high-intensity foreign signals. The examination of this revealed that most of the disturbances stem from a quasi-periodic signal generated by FBMs, exactly, from a very special type of fetal activity, which represents the rhythmic forward–backward motion of the diaphragm.

During the examinations, it was also revealed that the FBMs consist of 0.7–1.25-s long concatenated series of episodes, which are highly chaotic. Therefore, general methods are not suitable to study them. At first, the episode was described with some basic features [25]. These features are:

- The starting points (SPs) of the single breathing movement and the turning points, which indicate the relaxation of the diaphragm;
- The position of SPs relative to each other;
- The frequency spectrum of the phonographic signal;
- The dynamic nature of the FBM, which is determined by the accelerations and decelerations of the movements.

These features can also define a template in the initial state of the measurement. The meaningful parts of these templates are the rising time, the first and second halves of the episode, and the reciprocal of the short-term minimum zone at the starting point of the FBM.

## 2. Materials and Methods

**Figure 1:**
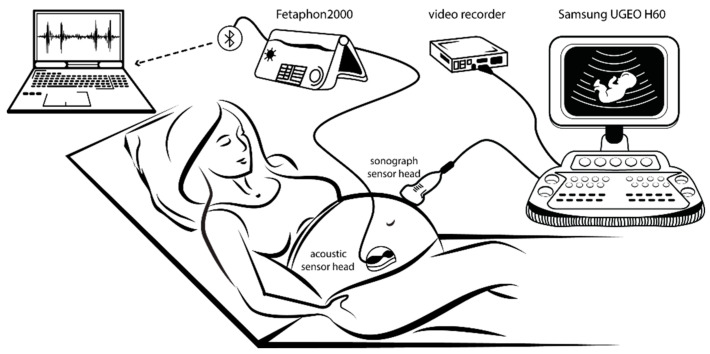
On the left side of the synchronous measurements set-up, it is visible that the phonographic (acoustic) sensor head is attached to the Fetaphon2000 monitor and its data are transferred via Bluetooth to a laptop. On the right side, the sonograph sensor head is attached to the ultrasound monitor used for validation only. The ultrasound measurements are recorded by a video recorder.

St. Margaret Hospital, Budapest, supported the research with medical consultations concerning more than 50 synchronous measurements using sonographic and phonographic recording (see Figure 1). The patients were informed about the purpose of this survey and they gave their consent to data collection. In this research, a novel investigation was set up to determine FBMs, which made the reproduction of previous measurements possible.

In the following, the usual terms are applied. According to this, one unit of an FBM is a so-called episode, and a series of concatenated episodes forms an epoch. The epochs can be divided into smaller episode groups, separated by shorter breaks.

These measurements were performed by expert sonographers and doctors. In the study, the ratio of investigated male to female fetuses was 50–50, and nearly 9000 FBM episodes were detected from the altogether 16-h long synchronous measurements. Based on these measurements, we were able to perform an accurate assessment of the FBMs. We found significant differences between male and female fetuses.

According to the biophysical profile (BPP) a 30-s long FBM epoch is prescribed during a 30-min long measurement [26]. Until now, these types of measurements have been accomplished by ultrasound sonography; however, this new method includes long-term measurements. The presented phonographic method is suitable for this purpose, providing more accurate detection of FBM episode lengths and strengths. When ultrasound does not provide a clear answer about fetal activity, these types of measurements may be important for the purpose of a presumed diagnosis. This is especially true in suspicious cases, where long-term monitoring is necessary.

### 2.1. Measuring Device

For the FBM measurements, the commercial Fetaphon2000 type phonocardiographic monitor was applied (its data are in Table 1). As it was optimized for fetal heart examinations, its lower frequency limit must be adapted to FBM detections.

The amplitude of the detected signals depends on the position of the fetus. Therefore, especially during longer measurements, the position of the sensor needs to be corrected occasionally. Furthermore, the automatic gain control also adjusts the amplification within a narrow range. Experience has shown that the intensity of the FBM signal is normally 3‒4 times larger than that of heart sounds, so this latter one can be utilized for setting the initial data range of the FBM measurements.

The acoustic data were transferred via Bluetooth to a laptop. A self-developed graphical user interface displayed the signal. This unit is also capable of handling the data of both the fetus and the mother; furthermore, annotations of different types of fetal activities were provided, e.g., hiccups, trunk rotation, FBMs, etc. The setup applied is illustrated on the left of Figure 1. It should be noted that since the sound to be recorded is coming from the diaphragm of the fetus, the acoustic sensing head must be adjusted to the maximum level of heart sound. The reason is that heart beats are continuous, thus providing a fixed point for the adjustment of the receiving head.

### 2.2. Validation of Data during the Research Phase

Figure 1 shows the measuring setup, but for the sake of simplicity, it has been completed with the devices used for the validation of data as well. These include a complete UGEO H60 type 3D Samsung sonographic system with a video recorder to collect image data (with 400 ms sampling time and 1366 × 768 × 24 bit resolution) of every movement of the fetal diaphragm. This will be used to verify the data obtained by the electric measurements. If a (not easy) photogrammetric process (Florido, [26]) is carried out on these images, we yield back practically the envelope of the measured phonogram signal, which expresses the intensity of the time function of the movements. Both data flows should be fully synchronous. This process perfectly shows the contraction and relaxation of the diaphragm, excluding any failure during the validation of the electrically measured movements.

### 2.3. Division of FBM Episodes into Sectors

For episodes with extremely irregular shapes, the previously used templates do not always give reliable results [25]. Therefore, to increase the accuracy of the measurements, we introduced a novel method that divides each episode into a number of narrow sectors. Based on our experiences, eight sectors are already enough to provide an accurate picture about the typical shape of the FBM, but this sector number is not too high either, so it does not significantly increase computing capacity.

**Figure 2:**
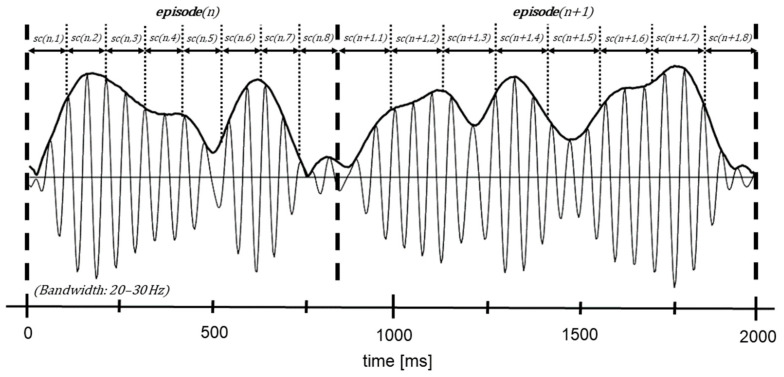
Divided episodes into eight equal sectors, where the episode length is different.

Figure 2 shows a sector division of two consecutive, but substantially different, episodes. Each sector is characterized by its intensity. Empirically, it was found that the most informative frequency band is between 20 and 30 Hz. Using more narrow frequency bands, the detection of FBM episodes can also be more accurate; however, the usage of multiple frequency bands can also significantly increase the real-time computing capacity [25]. The sector intensities are defined by sc(n,i), which is equal to the integrated Hilbert transform of a given sector:

(1) sc(n,i)=∫ℋ[s(n,i)]

where n refers to the index of episodes, i is the index of the sectors, and s(n,i) is the signal of the given sector.

### 2.4. Identification of FBM Episodes

During the measurement, an acoustic sensor was placed on the maternal abdominal wall where it makes audible the fetal heart sound as well as the movements. Based on these, the intensity of the heart signals can be determined, which is also related to the intensity of the FBMs. After all, as we wrote earlier, the intensity of an FBM is about 3–4 times higher than that of fetal heart sound, and this difference persists until the end of the third trimester, despite growth. Due to the histological location, the two acoustic signals change in a similar way.

The most characteristic episodes are the two, short time intervals with low-level signals which enclose a period with the maximum intensity one (see Figure 2). Based on this, we searched for two low-level signal intervals with 1 ± 0.25-s distance from each other, and between these, there is a high-intensity level period. To accept the episodes, the average intensity of previously determined heart sounds was also applied, namely the average value of fetal heart sound (*I_h_*). The low-intensity sectors must be lower than *I_h_*/2 value. Furthermore, the maximum value of the episode must be at least three times higher than the *I_h_* value.

**Remark**: If the intensity of the heart sounds is not available before beginning of the measurement (it may occur in the early third trimester), then it should be postponed. However, the *I_h_* value is primarily used only for the initialization of sector intensities.

After detection of potential episodes, the so-called weighted factors will help their final acceptance. Each sector has its own weighted factor, which is constantly updated according to the measurement data of the last 8–10 FBM episodes:

(2) $$w\left(n,i\right)=\frac{1}{K}\sum_{n-k}^{n-1}\frac{1}{sc\left(k,i\right)}$$

where *n* ‒ *k* are the indices of the accepted episodes for adjustment. The *k* < *K* region is the initializing time, where some starting factors should be applied—the most determining part of an episode is the last (8th) sector, giving the end of the former episode and, at the same time, the start of the next one. Using these factors for the initializing part, the obtained certainties will adjust the next K episodes, and so on. w(n,i) is continuously updated according to the previously accepted FBM episodes (see Equation (2)).

The product of the sc(n,i) sector intensity and the w(n,i) weighted intensity defines the c(n,i) certainty of the sectors, and the $C_{T}\left(n\right)$ certainty of the total episodes can be determined by summing the c(n,i) certainties. The c(n,i) and $C_{T}\left(n\right)$ certainties can be approved of if they reach 75% of the acceptance limits.

(3) c(n,i)≔sc(n,i)∗w(n,i)

(4) $$C_{T}\left(n\right)≔\sum_{i}^{M}c\left(n,i\right)$$

where M is the number of sectors. The sonographic measurements proved that the intensity of FBM episodes is directly proportional to the exerted force of the fetal diaphragm, and like fetal heart sounds, these movements will become more intense as the pregnancy progresses. Therefore, the weighted factors are dynamically changing, which is suitable for the continuous evaluation of the exerted force of the diaphragm. Furthermore, the sectors give a detailed picture of the episodes.

## 3. Results

Based on the examination of thousands of episodes, we could highlight novel aspects of FBMs compared to previous studies. The results are based on the signal shape, the intensity, the length of the episodes, the obtained episode groups, and the certainty evaluation. In the 20–30 Hz frequency band, we found that FBM epochs are formed by those episode groups which can be separated by short breaks. Based on the measurements, we have shown that the intensity of episodes significantly changes during the pregnancy, the number of short breaks between FBMs decreases, the episode groups become more and more contiguous, and that there are significant differences between male and female fetuses.

### 3.1. Intensity of FBM Episodes

Using the sectors, we obtained a much more nuanced image of the FBM episode from the phonogram signals. In this analysis, only the clear FBM episodes were inspected to obtain the fundamental characteristics of them. Based on sonographic validation measurements, the very uncertain episodes and the beginning and ending elements of episode groups were ignored.

**Figure 3:**
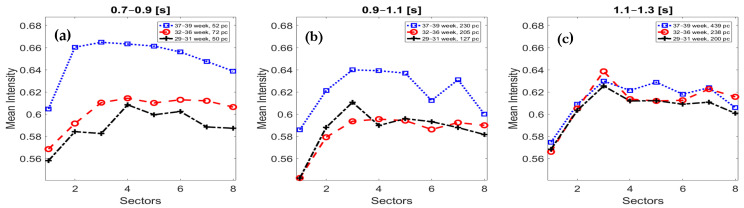
The sector intensity of the fetal breathing movement (FBM) episodes is depicted according to the gestational ages and the episode length. The subfigure (**a**) refers to the shorter episodes (0.7–0.9 s), (**b**) to the medium-sized (0.9–1.1 s) ones, and (**c**) shows the longer episodes (1.1–1.3 s). The lines mark the different gestational ages, where the black dashed line refers to 29–31 weeks, the red circled line means 32–36 weeks, and the blue squared line is between 37 and 39 weeks.

Because of these stricter conditions, several short FBMs were excluded from this study, especially episode groups that consist of 2‒3 members. Therefore, the number of observed episodes is drastically reduced compared to our previous surveys. Although these constraints may seem rigorous, it was essential to obtain the characteristics of “mature” FBMs. Fortunately, after this filtering, we still had enough data to be able to extract the main trends of FBMs (see Figure 3).

Figure 3 shows the sector intensities of FBM episodes according to the gestational ages and the episode length. The first remarkable fact is that the shorter episodes have a higher intensity at the end of the pregnancy. This fact refers to the rapid and powerful episodes as opposed to those mature FBMs that are slower. However, the rate of shorter episodes continuously decreases towards the end of the third trimester. Although it is not obvious, the data also suggest that fast diaphragm contraction requires more exertion, which is especially true at the end of the pregnancy (see the blue squared lines).

Table 2 shows the numerical results of Figure 3. The sectors’ intensity is getting higher in the first part of the episodes, which is decreased at the end part. The underlined and bold numbers mark the highest sector intensity volume of FBM episodes. Mainly, during the medium- and longer-sized episodes (see Figure 3b–c), an intermediate drop in intensity is clearly visible between the 4th and 6th sectors. The increased and decreased intensity refers to the diaphragmatic contraction and relaxation, respectively.

### 3.2. Length of FBM Episodes

Thousands of FBM episodes have been investigated to obtain an exact image about their ongoing changes. In the case of males, 4740 FBM episodes, while for females, 3100 episodes, were detected in the last trimester of the pregnancies. Although this issue has long been researched, FBMs have not yet been studied in such detail. Now, the main value of this study in contrast to the previous general FBM detection tests is that we can obtain a comprehensive assessment about the episode changes according to the different gestational ages and the sex of the fetus. Based on the large number of measurements, several previous assumptions have been proved.

**Figure 4:**
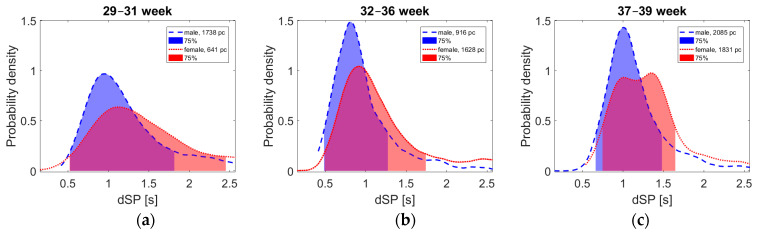
The probability densities of dSP time intervals according to the different gestational ages (see **a**–**c**) and the sex (blue marks the males and red marks the females). At the beginning of the third trimester, there are still many more uncertain FBM episodes than later during the pregnancy.

The FBM episodes were evaluated at different stages of the last trimester, which also showed significant differences according to sex (see Figure 4). It is important to emphasize that this research is still in its initial phase, but based on the number of detected FBM episodes, we can consider this data representative.

**Figure 5:**
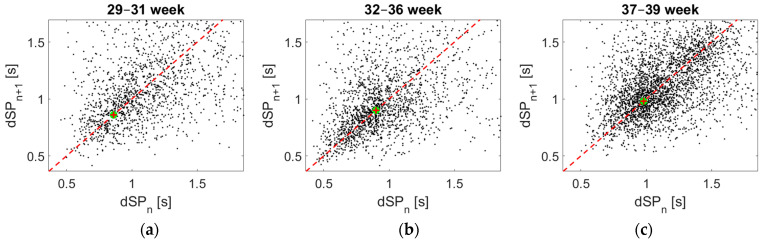
The time intervals of the FBM episodes in the well-known Poincaré-type recurrence plot according to the different gestational ages (see **a**–**c**). The distances of two neighboring starting points (dSPs) are very diverse, which became more and more homogeneous at the end of the last trimester.

We suppose that fatigue due to the physical effort of the fetus may be reflected in the FBM [27]. For this purpose, the total length of the measured episodes, called dSP (the distance of two neighboring SPs, including the distance of the minimum zone) has been determined as the function of the gestational age. The obtained data are given in Figure 5, represented on the well-known Poincaré-type recurrence plot in three different time groups of pregnancy weeks. However, it is important to note that dSP is not necessarily equal to the length of the episodes. In many cases, this length is the same, especially at the end of the pregnancy. Nevertheless, in many other cases, there is also a shorter break between the episodes, suggesting a momentary stop or rest of the fetuses.

Figure 5 displays groups standing from two consecutive episodes, placing (as points) the first one (dSP_n_) on the horizontal line and the second one on the vertical line. At equal time duration, this point is on the 45° line. Otherwise, the distance from this shows their difference. Evaluating the groups, one can see that with growing gestational age, the spread of the measured points decreases, showing that the breathing process will be more ordered or maybe more controlled by the fetus. Nevertheless, these all are moderately influenced by the spread of the break-zone’s length.

Since it is hard to reconstruct episode lengths visually from Figure 5, another form has been applied to display these lengths, shown in Figure 4, according to sexes. As one can see, the episode lengths increase here with the progressing number of weeks, resulting partly in the shortening of the break-zones. Another interesting point of this figure is the physiologically understandable (and also previously suspected) difference between the two sexes, since males are normally more muscular.

For both males and females, the standard deviation of dSP length is lower at 37–39 weeks of gestation. While long dSPs are common at the beginning of the third trimester, at the end of pregnancy, they are increasingly rare. This is because FBMs are more and more similar, homogeneous, and producing obviously fewer short breaks between the movements. Another remarkable fact is that the expected length of episodes is becoming longer in the last weeks. Furthermore, the different types of fetal movements can inhibit each other such as hiccup or body movements, which was also suggested by the sonographic measurements, as the different movements were very rarely detected simultaneously.

The double peak of female curves at 37–39 weeks is still unclear. Because this phenomenon did not occur obviously in the previous weeks, our hypothesis is that the length of these FBM episodes may increase significantly in the last stage of pregnancy. Since this study is in its initial stage, long-term conclusions can only be drawn from further measurements with respect to sex differences in relation to FBMs.

### 3.3. Certainty of the Measurement

**Figure 6:**
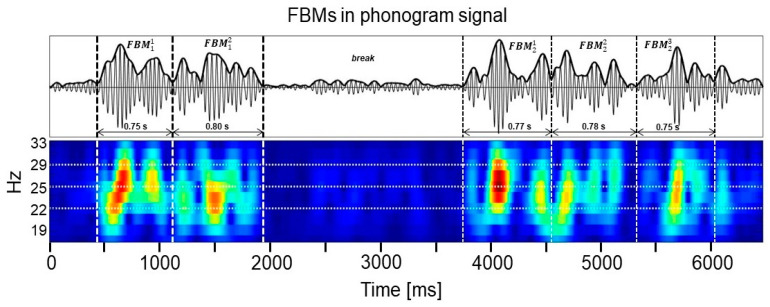
There is a time duration classified as break between two episode groups, displayed by wavelet transformation.

The main problem according to FBM measurements is the appearance of breaks, being either due to exhaustion or even for a simple pause. The intensity during this time will obviously be zero or at a very low level. Such a situation is shown on Figure 6, where the middle time period will be evaluated as a pause.

### 3.4. Stucture of FBM Episode Groups

During pregnancy, FBMs became more and more powerful, homogeneous, and contiguous. This section will discuss the structural development of FBM episode groups.

To evaluate the development of FBMs, 0.75–1.25-s long dSPs were investigated because they contain typical episodes. We also found several single episodes which were very uncertain FBMs. The proportion of contiguous episode groups increases as the pregnancy progresses, but the number and the rate of the longer episode groups is significantly higher in the case of males already from the beginning of the third trimester (see Table 3). Between weeks 29 and 31 of gestation, the average length of the longer-term groups was 14.4 ± 5.5 s for the 12 male episode groups. In contrast, at this age, for females, there are only two longer-term groups with 11.5 ± 2.1-s episode lengths. It is also very noteworthy that the proportion of the episodes which were classified into a minimally 10-member episode group was 19.7% for males and only 12.1% for females, even at the end of the third trimester.

However, these results may also lead to further conclusions, but further clinical measurements would be needed to surely confirm them. On the other hand, it can definitely be stated that FBMs are becoming more advanced at the end of pregnancy, and there is a significant difference between females and males with respect to FBMs.

## 4. Discussion

It was shown in the time-frequency map of the phonogram signal that FBM epochs can be divided into smaller episode group and separated by momentary breaks (see Figure 6), which may signal fetal fatigue. By the end of the third trimester, FBMs also became much more contiguous and quasi-continuous (see Figure 4). The shorter episodes reflect sudden exertions, and it is also observable that FBM intensity also increases in the last stage of the pregnancy (see Figure 3). This fact was also proved by the usage of the FBM episode sectors. This advanced assessment continuously adapts to environmental changes too. The number of long FBM episodes increases as gestational age increases, and the FBMs also become more certain for mature fetuses (see Figure 6). The FBM characteristics also significantly change during the pregnancy. In Table 3, the measurement results are remarkable, which also show significant differences between the male and female fetuses. Future research is promising in this field.

At the beginning of the third trimester, FBMs are quite rudimentary, but they are also very common. Because of the possibility of preterm birth, this period is the most critical one. That is why it would be necessary to obtain much more information about the physiological background of the fetuses in this period, in which the FBMs also play an important role.

Unfortunately, in some cases, it is difficult or impossible to measure the FHR well, but in this case, the measurement must be repeated after a short break. Fortunately, however, this phenomenon is not typical at the end of the third trimester. Although the FBM can be measured independently of the FHR, a reference signal intensity is required at the start of the measurement which provides baseline values. The great advantage of phonography is that it can measure not only the length of the episodes but also their intensity very accurately. We still have limited medical knowledge about FBM intensity progress, but the research is promising in this field.

It is also known that parasympathetic activity is increased by FBMs [28]. The absence of FBMs can be a pre-indicator of serious problems, e.g., intrauterine growth retardation (IUGR) or sudden infant death syndrome (SIDS), which might be prevented. In the case of IUGR, the fetal movements become slower and more monotonous as their variability in strength and amplitude is reduced [29,30,31]. Nearly 10% of newborn babies are affected by IUGR, where the major risks are preterm birth and respiratory insufficiency [32]. Furthermore, for sheep fetuses, it has already been confirmed that a lack of FBM can also be the sign of placental insufficiency [33]. This research tends to highlight the importance of FBM monitoring to avoid unexpected intrauterine deaths.

Due to more and more delayed childbearing, there is an increasing risk factor in the third trimester, but many of the new risks are still unknown. Today, telemedicine-based fetal monitoring is not widespread, but with the development of the available technologies, it will become a more prominent part of our lives. In the near future, the developed phonographic method can be a solution for that, which provides unlimited measurements. It is a low-cost and reliable assessment method that makes a more efficient monitoring of fetal activity possible, offering a new opportunity to determine FBMs safely, even at home.

It also turned out that detailed evaluation of each episode can provide additional information about the development of the diaphragm and the respiratory nervous system. It must be noted, however, that despite the huge amount of available data and clinical experience, researchers’ understanding of the FBM mechanisms is still far from being accurate. The present study represents a huge step forward on this road towards the exploration and understanding of FBMs. Although this research is based on decades of experience, the potential for the application of telemedicine in this area is, as yet, still untapped.

## 5. Conclusions

Our comprehensive survey analyzed approximately 16 h of synchronous recordings of fetal tests taken in the third trimester of pregnancy. It became clear that the characteristics of FBM change over time—movements become longer and more intense towards the end of pregnancy. Furthermore, it was revealed that the FBMs of female and male fetuses differ considerably both in their length and the intervals between them. These novel results shed light on the properties of FBMs, which can pave the way for a more accurate testing method and can also help in setting up the criteria for normal and abnormal FBMs. By detecting abnormal FBMs, the lives of several fetuses can be saved in the future.

The certainty of FBM measurement (such as selectivity, specificity, etc.) from the point of view of the BPP is extremely uncertain because FBMs occur sporadically. However, we are sure that by the introduction of the presented phonographic method, the prescribed data for FBM measurements will be more definite.

## Figures and Tables

**Table 1:** Specification of Fetaphon2000 fetal heart monitor.

Type of Data	Sampling Time	Data Resolution	Bandwidth	Electric Power
Sound	3 ms	8 bit	15–100 Hz	9 V_DC_/50 mA

**Table 2:** The average sector intensity volume of FBM episodes.

gAge	Length	No. Episodes	Sector(1)	Sector(2)	Sector(3)	Sector(4)	Sector(5)	Sector(6)	Sector(7)	Sector(8)
**29–31 weeks**	**0.7–0.9 s**	**50 pc**	0.558	0.584	0.583	0.608	0.599	0.602	0.588	0.587
	**0.9–1.1 s**	**127 pc**	0.542	0.588	0.611	0.590	0.596	0.593	0.588	0.582
	**1.1–1.3 s**	**200 pc**	0.568	0.603	0.625	0.612	0.612	0.609	0.611	0.601
**32–36 weeks**	**0.7–0.9 s**	**72 pc**	0.569	0.592	0.610	0.614	0.610	0.613	0.612	0.606
	**0.9–1.1 s**	**205 pc**	0.542	0.579	0.594	0.596	0.594	0.586	0.592	0.590
	**1.1–1.3 s**	**238 pc**	0.566	0.605	0.639	0.614	0.612	0.613	0.623	0.616
**37–39 weeks**	**0.7–0.9 s**	**52 pc**	0.605	0.660	0.665	0.663	0.661	0.656	0.648	0.639
	**0.9–1.1 s**	**230 pc**	0.586	0.621	0.640	0.639	0.637	0.612	0.631	0.600
	**1.1–1.3 s**	**439 pc**	0.575	0.609	0.630	0.621	0.629	0.618	0.624	0.606

**Table 3:** The FBM episode groups’ formation during the different gestational ages in the last trimester at males and females fetuses.

	No. Episode Groups (pc)			No. Episodes (pc)			Percentage Distribution of Episodes (%)			Mean ± SD No. Episodes (pc)		
Weeks	29–31	32–36	37–39	29–31	32–36	37–39	29–31	32–36	37–39	29–31	32–36	37–39
**MALE**												
**1 episode**	613	302	480	613	302	480	35.3	33.0	23.1	1	1	1
**2–5 episode**	283	125	301	777	336	833	44.7	36.7	40.0	2.7 ± 1	2.7 ± 0.9	2.8 ± 1
**6–10 episode**	26	14	51	176	94	359	10.1	10.3	17.2	6.7 ± 1	6.7 ± 0.7	7 ± 1
**10< episode**	12	12	26	171	183	410	9.8	20.0	19.7	14.3 ± 5.5	15.3 ± 5.9	15.8 ± 8.2
**FEMALE**												
**1 episode**	323	555	793	323	555	793	50.7	34.1	43.4	1	1	1
**2–5 episode**	99	241	202	249	665	563	39.1	40.9	30.8	2.5 ± 0.9	2.8 ± 1	2.8 ± 1
**6–10 episode**	6	41	37	42	293	251	6.6	18.0	13.7	7 ± 0.9	7.2 ± 1.1	6.8 ± 1
**10< episode**	2	9	14	23	114	222	3.6	7.0	12.1	11.5 ± 2.1	12.7 ± 3.6	15.9 ± 10.9

## Data Availability

Data sharing not applicable.

## Abbreviations

AGC	Automatic gain control
BPP	Biophysical profile
dSP	The distance of two neighboring SPs
FBM	Fetal breathing movement
FHR	Fetal heart rate
IUGR	Intrauterine growth retardation
SIDS	Sudden infant death syndrome
SP	Starting point of FBM episode
